# A transcription factor signature predicts the survival of patients with adrenocortical carcinoma

**DOI:** 10.7717/peerj.12433

**Published:** 2021-12-09

**Authors:** Jianyu Zhao, Bo Liu, Xiaoping Li

**Affiliations:** 1 Department of Endocrinology, China-Japan Union Hospital of Jilin University, Changchun, Jilin, China; 2 Department of Pediatrics Endocrinology, The First Hospital of Jilin University, Changchun, Jilin, China, Jilin, China

**Keywords:** Adrenocortical carcinoma, TCGA, GEO, Transcription factor, Prognosis

## Abstract

**Background:**

Adrenocortical carcinoma (ACC) is a rare endocrine cancer that manifests as abdominal masses and excessive steroid hormone levels and is associated with poor clinical outcomes. Transcription factors (TFs) deregulation is found to be involved in adrenocortical tumorigenesis and cancer progression. This study aimed to construct a TF-based prognostic signature for the prediction of survival of ACC patients.

**Methods:**

The gene expression profile and clinical information for ACC patients were downloaded from The Cancer Genome Atlas (TCGA, training set) and Gene Expression Omnibus (GEO, validation set) datasets after obtained 1,639 human TFs from a previously published study. The univariate Cox regression analysis was applied to identify the survival-related TFs and the LASSO Cox regression was conducted to construct the TF signature based on these survival-associated TFs candidates. Then, multivariate analysis was used to reveal the independent prognostic factors. Furthermore, Gene Set Enrichment Analysis (GSEA) was performed to analyze the significance of the TFs constituting the prognostic signature.

**Results:**

LASSO Cox regression and multivariate Cox regression identified a 13-TF prognostic signature comprised of CREB3L3, NR0B1, CENPA, FOXM1, E2F2, MYBL2, HOXC11, ZIC2, ZNF282, DNMT1, TCF3, ELK4, and KLF6. The risk score based on the TF signature could classify patients into low- and high-risk groups. Kaplan-Meier analyses showed that patients in the high-risk group had significantly shorter overall survival (OS) compared to the low-risk patients. Receiver operating characteristic (ROC) curves showed that the prognostic signature predicted the OS of ACC patients with good sensitivity and specificity both in the training set (AUC > 0.9) and the validation set (AUC > 0.7). Furthermore, the TF-risk score was an independent prognostic factor.

**Conclusions:**

Taken together, we identified a 13-TF prognostic marker to predict OS in ACC patients.

## Introduction

Adrenocortical carcinoma (ACC) is a rare endocrine cancer with an annual incidence of 0.7–2.0 cases per million (Bilimoria et al., 2008; Else et al., 2014). It usually affects adults aged around 40–50 years and children younger than 10 years (Koschker et al., 2006; Rodgers et al., 2006). Clinical manifestations of ACC include abdominal masses and elevated steroid hormones and result in overall poor outcomes with 5-year survival ranging from 32% to 45% (Icard et al., 2001). Therefore, it is essential to identify prognostic markers of ACC to screen for patients at high risk.

Transcription factors (TFs) are regulatory proteins that bind to the promoter sequences of genes and decrease or increase their transcription (Latchman, 1997), and thus control cell differentiation (Arendt et al., 2016), proliferation (Bouchard, Staller & Eilers, 1998), and death (Shaulian & Karin, 2002). Given the important role of TFs in determining cell fate, the contribution to tumor development and progression is easily made. Not surprisingly, the genes encoding TFs are often aberrantly expressed in ACC (Brown et al., 2018), and the relationship between the TFs and survival has been implicated in ACC patients (Parviainen et al., 2013b). For instance, Snail is overexpressed in numerous ACC patients and associated with decreased survival (Waldmann et al., 2008). In addition, TGF-β pathway components including GATA-6 and SF-1 are also correlated with poor outcomes in ACC patients (Parviainen et al., 2013a). A recent study has shown that high expression of HOXB9 is associated with a poorer prognosis of ACC patients (Francis et al., 2021). Although these reports demonstrated that the TFs are closely correlated with the prognosis of ACC patients, fewer studies have investigated the prognostic value of the TF signature in ACC patients. Therefore, it is necessary to investigate the correlation between the TFs and the survival of ACC patients and construct a prognostic TF signature for predicting the survival of ACC patients.

The Cancer Genome Atlas (TCGA) and Gene Expression Omnibus (GEO) datasets have enabled researchers to correlate clinical outcomes with transcriptomic profiles. To this end, we systematically analyzed the gene expression data of ACC datasets using univariate and multivariate Cox regression models. Based on the survival analysis, we then developed a 13-TF prognostic signature and validated this model in an independent microarray data set from GEO.

## Materials and Methods

### Data collection and preprocessing

A total of 1,639 human TFs were identified from a previously published study (Lambert et al., 2018). TCGA ACC level 3 RNAseqv2 data (RSEM_genes_normalized file) and corresponding clinical information were downloaded from the TCGA database (https://tcga-data.nci.nih.gov/). A total of 79 patients with ACC from TCGA were included after excluding those lacking complete clinical and survival data, which served as training sets. The gene expression profiling of 22 patients with ACC was selected *via* the accession number GSE19776 from GEO (http://www.ncbi.nlm.nih.gov/geo/) using the GPL570 platform [HG-U133_Plus_2] Affymetrix Human Genome U133 Plus 2.0 Array, which served as validation set. The clinical data included age, gender, and tumor stage. Firstly, we obtained 1,554 overlapped TFs between TFs in literature and TCGA dataset, and 1,508 overlapped TFs between TFs in literature and GEO dataset. A total of 118 TFs were excluded from the TCGA dataset by removing lower expression genes. Next, we acquired the common TFs between the TCGA and GEO datasets.

### Survival analysis

After obtained common TFs between TCGA and GEO datasets, univariate Cox survival analysis was performed using Cox proportional hazards regression model. Only TFs with Cox *P* < 0.001 and Log Rank *P* < 0.001 on univariate analysis were incorporated into the Lasso Cox regression analysis. Kaplan-Meier method was used to analyze the correlation between overall survival (OS) and TF expression, and the OS of different patient groups were compared using the Log-Rank test. The “survival” package (Therneau & Lumley, 2015) in R software was used for survival analysis and the time-dependent ROC (Receiver Operating Characteristic) curve analysis was performed using the “survival ROC” package (Robin et al., 2011).

### Construction of ACC prognostic signature

LASSO Cox regression model was widely used for high-dimensional predictor identification. In the present study, OS-associated TFs were used to select the significant TFs associated with the OS of ACC patients according to the coefficient value. These factors were incorporated in the multivariate Cox regression model to construct the ACC prognostic signature. The risk score for each TF-encoding gene was calculated as follows: $Risk{\rm \; }score{\rm \; } = \mathop \sum \nolimits_{i = 1}^n ex{p_i}\ {\rm *}\ {\beta _i}$, where n is the number of selected genes, exp_i_ is the expression level of gene i, and β_i_ is the coefficient of gene i.

### mRNA expression of 13 TFs in ACC cell line

Gene expression data of all 13 TFs in the prognostic signature from the SW13 cell line were downloaded directly from the Cancer cell line encyclopedia (CCLE) database (https://portals.broadinstitute.org/ccle/home), which is an open-access database covering large-scale deep sequencing raw data of more than 900 human cancer cell lines.

### Gene set enrichment analysis (GSEA)

GSEA was performed to analyze the significance of the 13 TFs constituting the prognostic signature using GSEA v2.0.12 (http://www.broadinstitute.org/gsea) by computing the enrichment score for each gene set (Subramanian et al., 2005). The distributions of these TFs against the rank-ordered gene ontology (GO) hallmarks were characterized using GSEA with the default settings. Positive and negative normalized scores indicated enrichment in the high-risk and low-risk groups respectively.

## Results

### Construction of the TF prognostic signature

**Figure 1:**
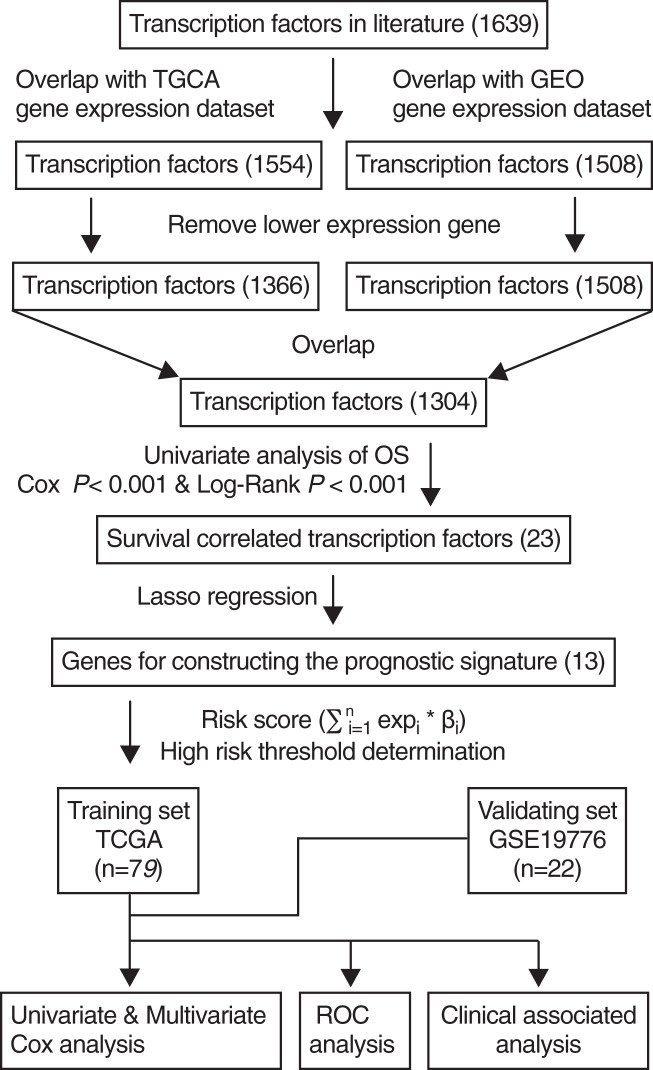
Flowchart describing the construction and validation of the 13-TF prognostic signature.

The scheme for developing the TF signature is outlined in Fig. 1. After initially identifying 1,639 TFs by literature search, the down-regulated genes were removed and 1,304 common TFs were screened from TCGA and GEO datasets. Using the TCGA training set (*n* = 79), univariate regression analysis showed that 23 TFs were correlated with OS (Cox-*P* < 0.001 and Log-Rank *P* < 0.001), of which 13 were identified by the Lasso regression analysis, and the risk score was calculated by multivariate cox analysis.

**Figure 2:**
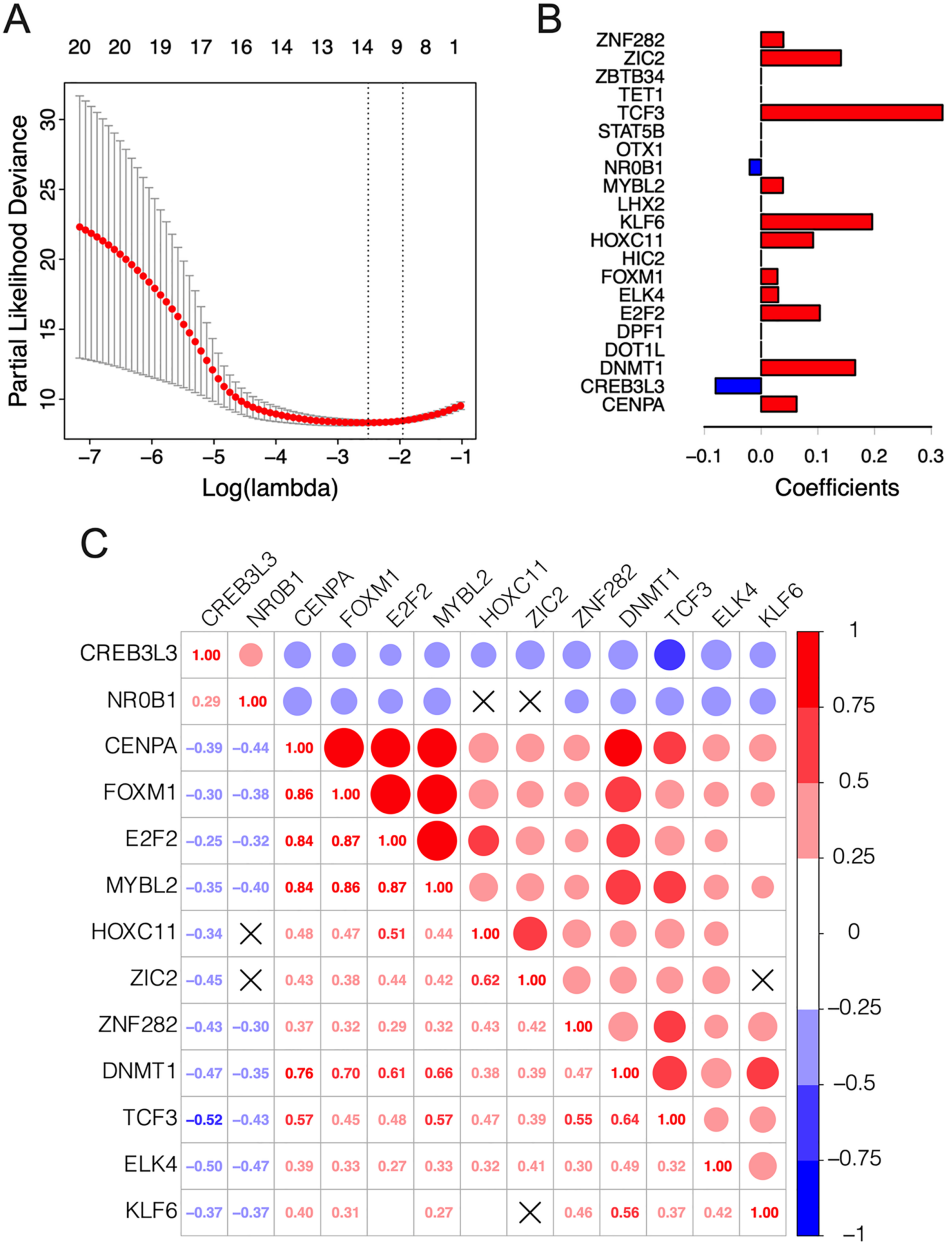
Construction of the 13-TF prognostic signature. (A) Selection of optimal tuning parameter (λ) in the LASSO model. The dotted vertical lines are drawn at the optimal values by minimum criteria (left) and 1-SE criteria (right). Error bars represent standard error (SE); (B) coefficient of each transcription factor; (C) Spearman correlation analysis for the 13 TFs. Red and green respectively represent the positive and the negative correlation. The size of the point represents the strength of correlation. The TFs crossed mean *P* > 0.05.

**Figure 3:**
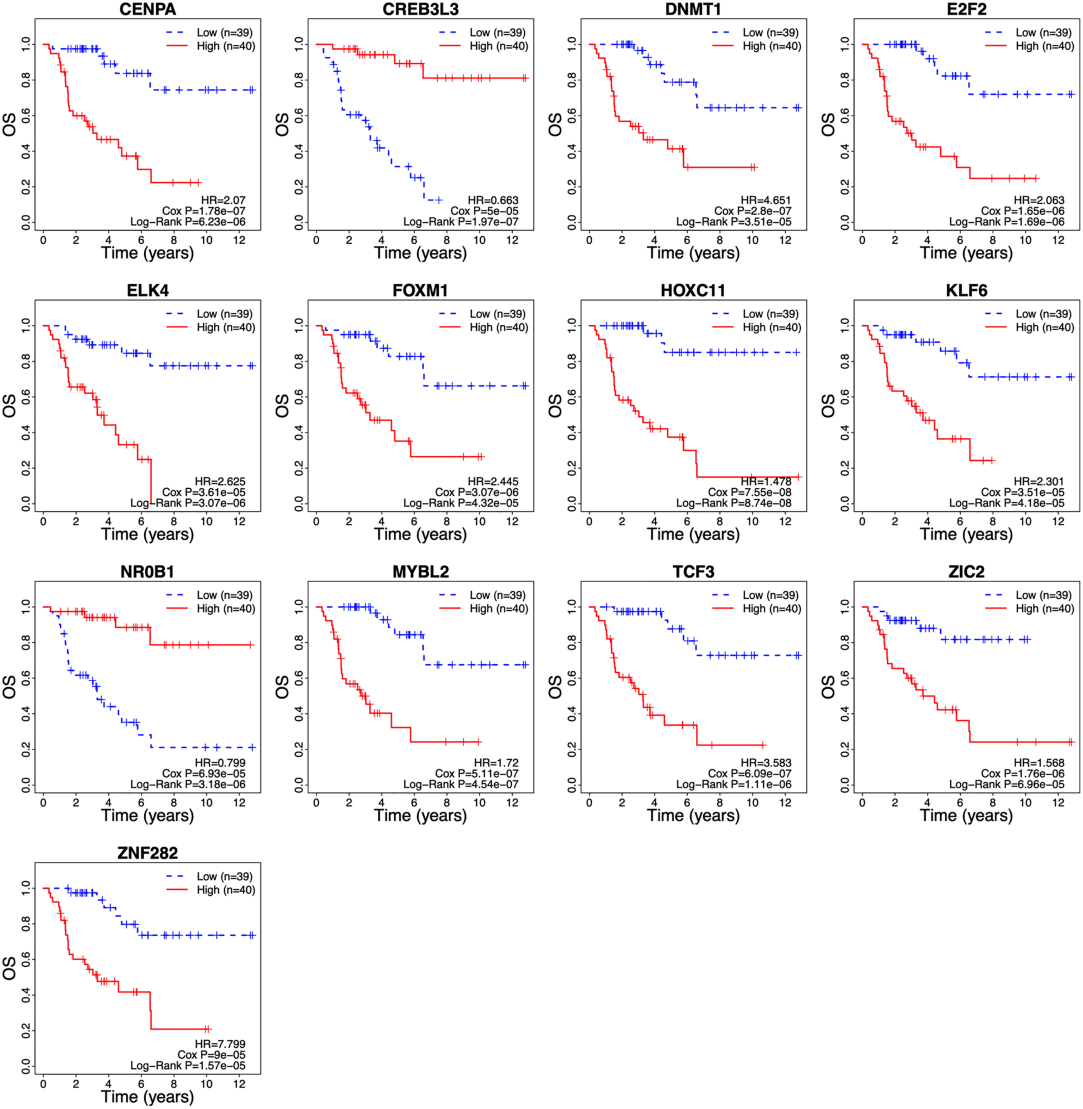
Survival curves of ACC patients demarcated on the basis of TF risk score. The median risk score was used as the cutoff value. ACC, Adrenocortical carcinoma; OS, overall survival.

The λ value was selected in the LASSO Cox regression analysis when the median of the sum of squared residuals was the smallest (Fig. 2A). The following survival-related TFs with non-zero coefficients were then screened: CREB3L3 (cAMP-responsive element-binding protein 3-like 3), NR0B1 (nuclear receptor subfamily 0, group B, member 1), CENPA (centromere protein-A), FOXM1 (Forkhead Box M1), E2F2 (E2F transcription factor 2), MYBL2 (v-myb myeloblastosis viral oncogene homolog (avian)-like 2), HOXC11 (homeobox C11), ZIC2 (Zic family member 2), ZNF282 (zinc finger protein 282), DNMT1 (DNA methyltransferase 1), TCF3 (transcription factor 3), ELK4 (ETS transcription factor ELK4) and KLF6 (Krüppel-like factor 6) (Fig. 2B). Only CREB3L3 and NR0B1 were negatively correlated with the remaining TFs (Fig. 2C), while CENPA, FOXM1, and E2F2 displayed a strong correlation (Table 1). As shown in Fig. 3A, a total of 79 patients from the TCGA database were divided into a high-expression group (*n* = 40) and a low-expression group (*n* = 39). Significantly, high expression of CREB3L3 (HR = 0.663, Cox *P* = 5e−05, Log-Rank *P* = 1.97e−07) and NR0B1 (HR = 0.799, Cox *P* = 6.93e−05, Log-Rank *P* = 3.18e−06) were associated with good prognosis, and that of other TFs with poor prognosis (HR > 1, *P* < 0.001).

**Table 1:** Information of the 13 transcription factors for constructing the prognostic signature.

Gene symbol	Stable ID	Gene type	Chr position (start–end)
CENPA	ENSG00000115163	Protein coding	2 (26764289–26801067)
CREB3L3	ENSG00000060566	Protein coding	19 (4153631–4173054)
DNMT1	ENSG00000130816	Protein coding	19 (10133345–10231286)
E2F2	ENSG00000007968	Protein coding	1 (23506438–23531233)
ELK4	ENSG00000158711	Protein coding	1 (205597556–205631962)
FOXM1	ENSG00000111206	Protein coding	12 (2857681–2877155)
HOXC11	ENSG00000123388	Protein coding	12 (53973126–53977643)
KLF6	ENSG00000067082	Protein coding	10 (3775996–3785281)
MYBL2	ENSG00000101057	Protein coding	20 (43667019–43716495)
NR0B1	ENSG00000169297	Protein coding	X (30304206–30309598)
TCF3	ENSG00000071564	Protein coding	19 (1609290–1652615)
ZIC2	ENSG00000043355	Protein coding	13 (99981784–99986765)
ZNF282	ENSG00000170265	Protein coding	7 (149195546–149226238)

**Note:**

Chr, chromosome.

### TF signature risk score can predict prognosis of ACC patients

**Figure 4:**
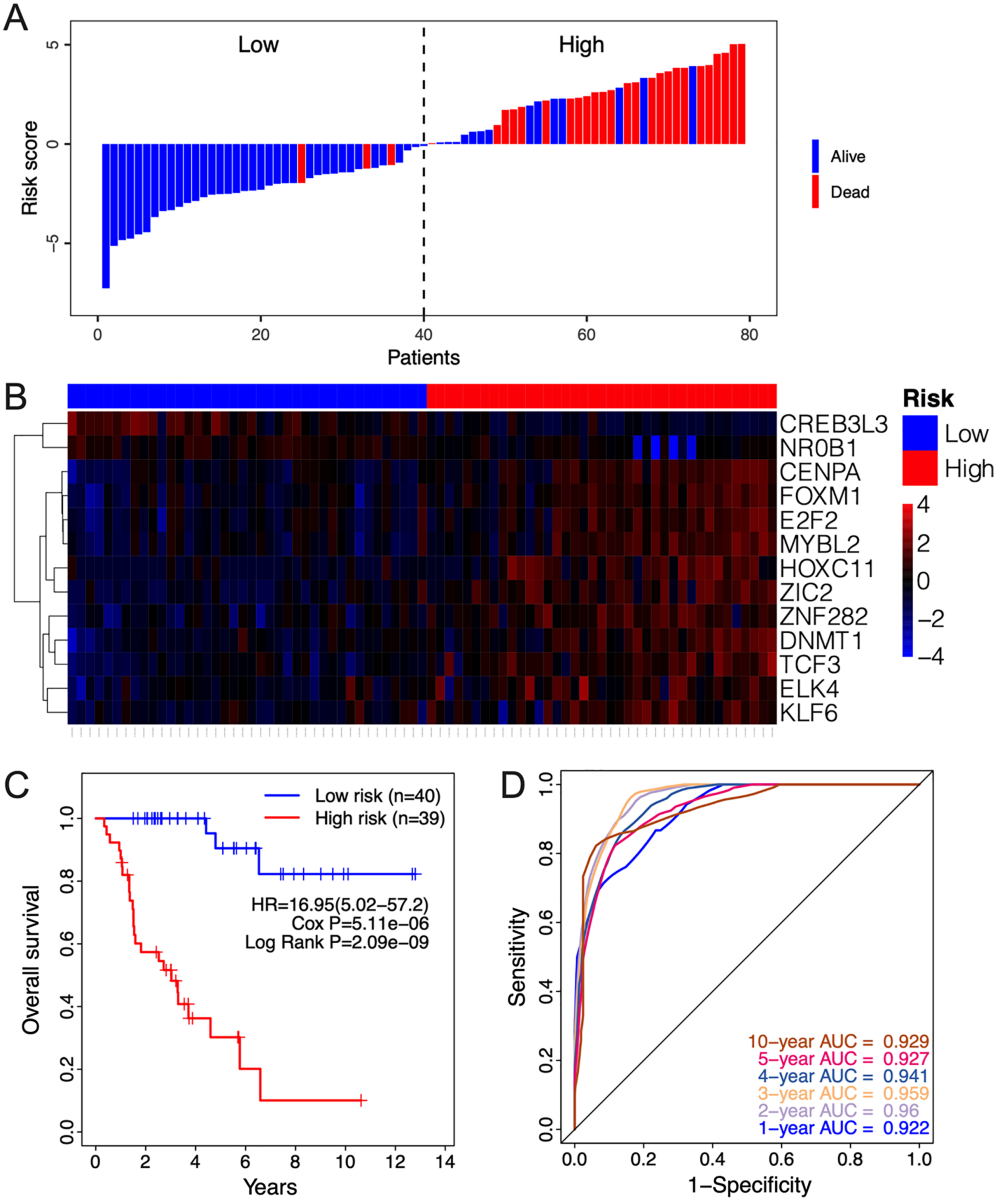
Risk score calculated by the 13-TF signature and Kaplan-Meier survival in TCGA dataset. (A) Risk score distribution of the 13-TF signature and patient survival status; (B) heatmap of each TF; (C) Kaplan-Meier survival plots; (D) prognostic accuracy of the 13-TF signature. HR, Hazard ratio; AUC, area under the curve.

**Figure 5:**
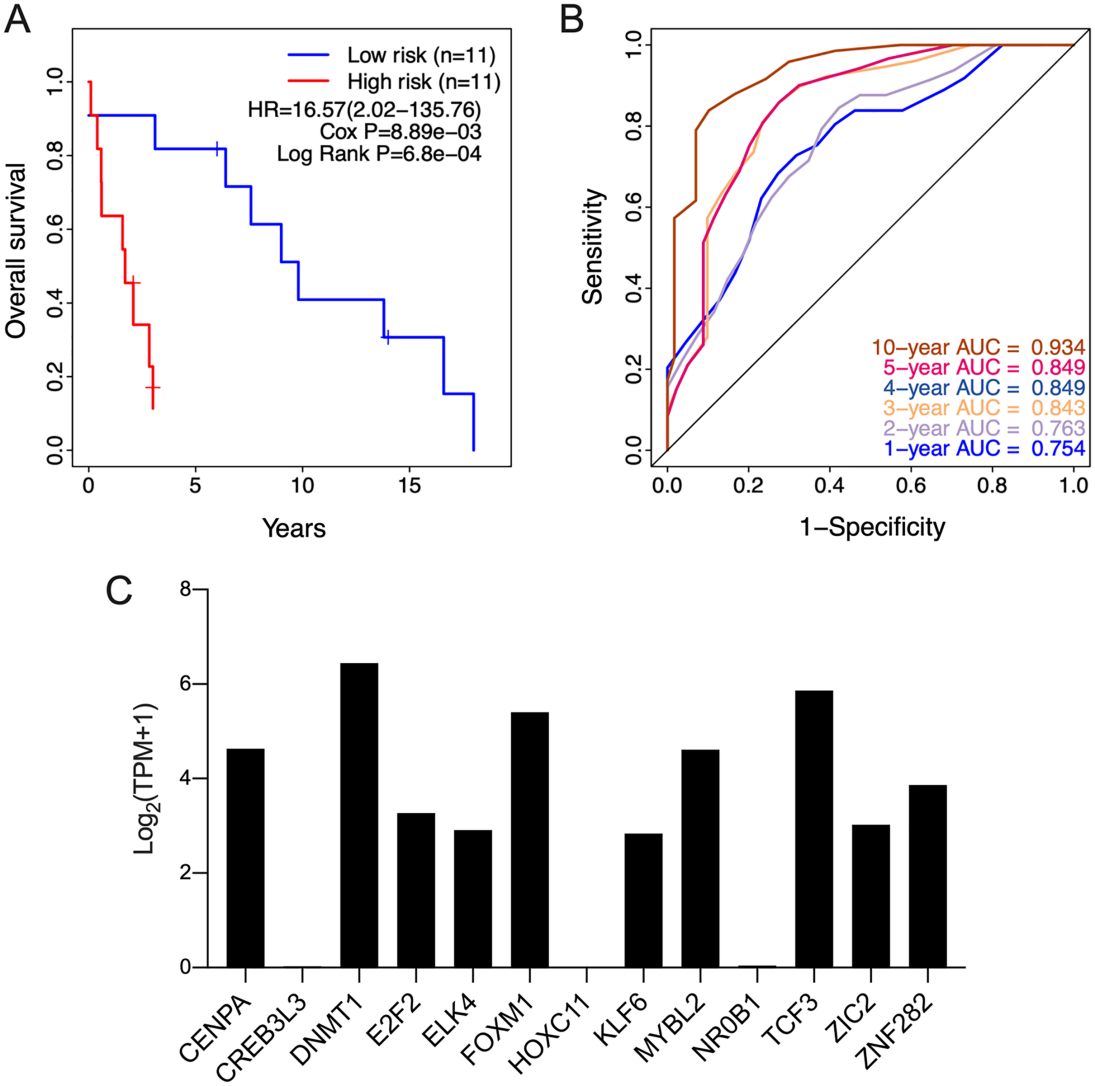
Validation of the 13-TF signature in the GEO (Gene Expression Omnibus) dataset and the CCLE (Cancer cell line encyclopedia) database. (A) Kaplan-Meier plots for OS of ACC patients in GEO dataset. (B) ROC curve for predicting OS at different time points. (C) mRNA levels of 13 TFs in SW13 cells from CCLE database. HR, Hazard ratio; ROC, receiver operating characteristic; AUC, area under the curve; TPM, transcripts per million.

The clinical relevance of these TFs was further assessed by multivariate Cox regression analysis based on the TCGA training set, and the risk score based on their expression levels and coefficients was calculated. The 13-TF risk score classified the patients from TCGA training set into the high- (*n* = 39) and low-risk (*n* = 40) groups (Fig. 4A). Except for CREB3L3 and NR0B1, all TFs were overexpressed in the high-risk group (Fig. 4B). Furthermore, ACC patients in the high-risk group had significantly shorter survival compared to the low-risk patients (HR = 16.95 (5.02–57.2); Cox *P* = 5.11e−06; Log Rank *P* = 2.09e−09) (Fig. 4C). The sensitivity and specificity of the 13-TF signature were determined using time-dependent receiver operating characteristic (ROC) analysis, and the area under curves (AUCs) at all follow-up time points were greater than 0.9 (Fig. 4D). The predictive model was then validated in a GEO dataset, and as shown in Fig. 5A, the high-risk group (*n* = 11) had worse survival compared to the low-risk group (*n* = 11). In addition, the AUC values of the signature were greater than 0.75 (Fig. 5B). Next, we investigated the mRNA levels of all 13 TFs in the ACC cell line through the CCLE database (Fig. 5C). The results showed that the mRNA levels of CREB3L3, NR0B1, and HOXC11 were almost undetectable in SW13 cells. In contrast, the other 10 TFs were highly expressed in ACC cells. Taken together, the 13-TF signature can predict the survival of ACC patients with high sensitivity and specificity.

### The risk score is an independent prognostic factor of ACC

**Figure 6:**
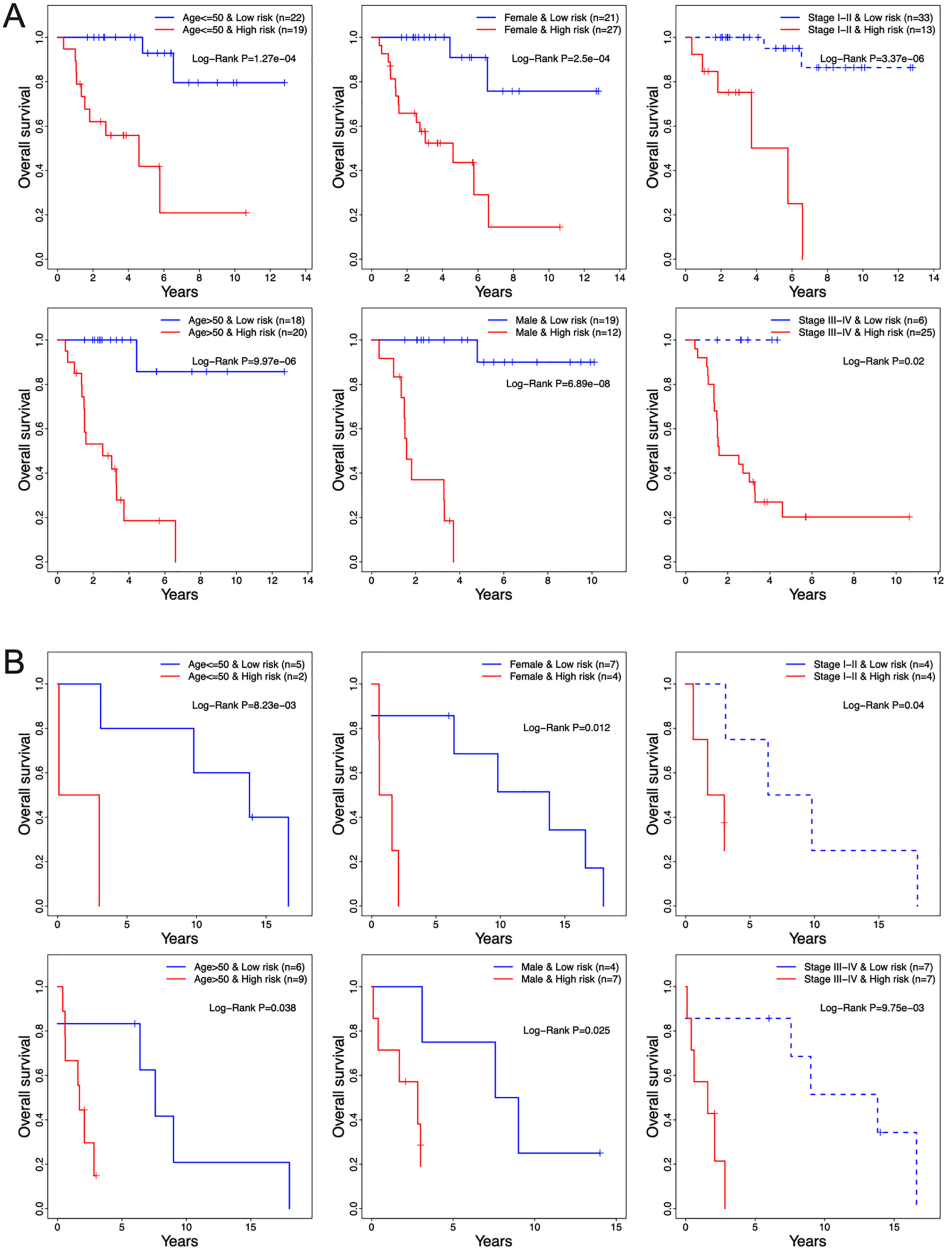
Clinical characteristics of TCGA and GEO cohorts stratified by the 13-TF risk score. (A) TCGA set; (B) GEO set. TCGA, The Cancer Genome Atlas; GEO, Gene Expression Omnibus.

The prognostic relevance of the 13-TF signature was further validated by multivariate Cox regression analysis after normalizing for variables including age, gender, and pathological stage. In both the training and validation ACC cohorts, the 13-TF risk score was an independent prognostic factor (Table 2). However, no significant correlation was seen between OS and age, gender, or pathological stage. The high- and low-risk groups of both training and validation datasets were further divided into subgroups based on age (≤50 *vs* >50 years), gender (male *vs* female), and pathological stage (I–II *vs* III–IV). As shown in the Figs. 6A and 6B, patients in the high-risk group had poor prognosis and significantly shorter survival compared to those in the low-risk group regardless of other variables. Thus, the 13-TF signature is an independent prognostic predictor of ACC.

**Table 2:** Cox regression analysis of the correlation between clinicopathological factors and overall survival of ACC patients.

Variables	Group	Patients (N)	Univariate analysis		Patients (N)	Multivariate analysis	
			HR (95% CI)	*P*		HR (95% CI)	*P*
**TCGA**							
Risk score	Low/High	40/39	16.95 [5.02–57.2]	5.11E−06	39/38	22.59 [4.55–112.22]	1.38E−04
Age	<=50/>50	41/38	1.8 [0.85–3.82]	1.27E−01	40/37	1.47 [0.63–3.45]	3.73E−01
Gender	F/M	48/31	1.00 [0.47–2.14]	9.99E−01	48/29	1.96 [0.79–4.84]	1.46E−01
Stage	I-II/III-IV	46/31	6.48 [2.71–15.5]	2.72E−05	46/31	1.78 [0.72–4.39]	2.13E−01
**GSE19776**							
Risk score	Low/High	11/11	16.57 [2.02–135.76]	8.89E−03	11/11	18.85 [2.09–170.08]	8.89E−03
Age	<=50/>50	15/7	1.72 [0.60–4.88]	3.11E−01	15/7	1.23 [0.38–3.97]	7.33E−01
Gender	F/M	11/11	1.26 [0.46–3.44]	6.50E−01	11/11	0.77 [0.26–2.26]	6.32E−01
Stage	I-II/III-IV	14/8	1.21 [0.44–3.29]	7.12E−01	14/8	1.46 [0.51–4.14]	4.81E−01

**Note:**

HR, Hazard ratio; CI, Confidence interval.

### The 13-TF signature is associated with cancer-related functions

**Figure 7:**
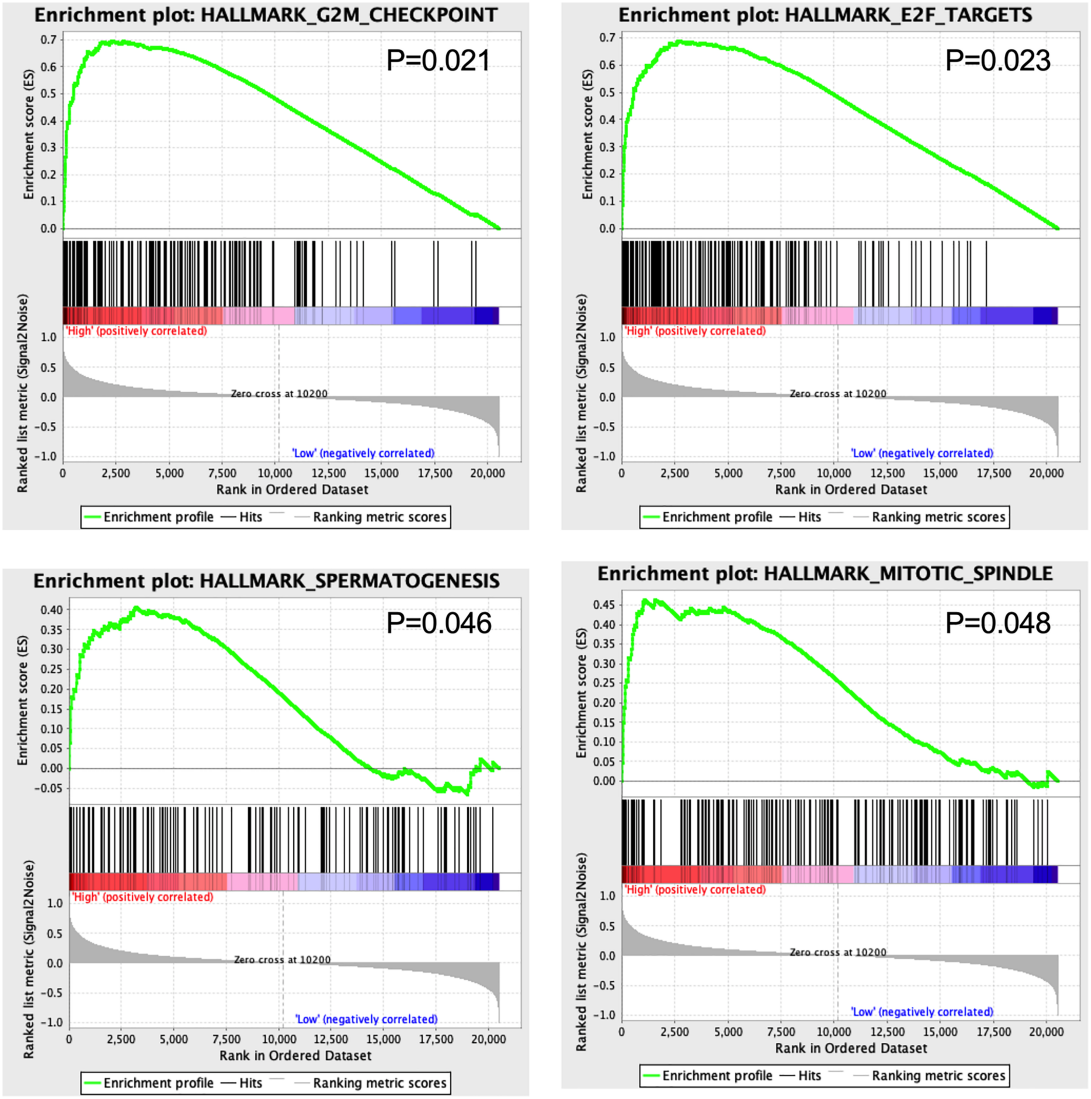
Gene set enrichment analysis of 13 survival-related TFs.

GSEA results showed that four hallmarks including G2M_CHECKPOINT (*P* = 0.021), E2F_TARGETS (*P* = 0.023), SPERMATOGENESIS (*P* = 0.046), and MITOTIC_SPINDLE (*P* = 0.048) were significantly enriched in high-risk patients, suggesting a mechanistic basis of the prognostic role of the 13-TF signature in ACC (Fig. 7).

## Discussion

ACC is a rare endocrine cancer with limited therapeutic options and poor clinical outcomes. Studies have increasingly identified molecular diagnostic and prognostic signatures of various cancers by screening multiple databases *via* high-throughput technologies such as microarrays and next-generation sequencing. Transcription factors (TFs) are often aberrantly expressed in tumors, and correlated with cancer prognosis (Lutz, Leon & Eilers, 2002; Ozaki & Nakagawara, 2011). Recent studies have identified specific TFs that are independent prognostic factors in various cancers (Jiang et al., 2010; Span et al., 2002; Wolf et al., 2005). The zinc-finger transcription factor Snail is associated with decreased survival of ACC patients and a higher risk of distant metastasis (Waldmann et al., 2008). However, a TF-related prognostic signature has not yet been identified for ACC.

We analyzed the gene expression profiles of ACC patients deposited in GEO and TCGA databases and constructed a prognostic signature of 13 TFs, including CREB3L3, NR0B1, CENPA, FOXM1, E2F2, MYBL2, HOXC11, ZIC2, ZNF282, DNMT1, TCF3, ELK4, and KLF6. NR0B1, also known as DAX1, is an atypical orphan nuclear hormone receptor that is expressed in the adrenal glands and at all levels of the hypothalamo-pituitary-gonadal axis (Guo et al., 1996). Previous studies have shown that NR0B1 was associated with a variety of cancers, although its role in promoting or suppressing tumors is not consistent (Ranhotra, 2013). NR0B1 silencing decreased the *in vitro* invasiveness of the lung cancer cell line A549 and inhibited xenograft growth without affecting cell proliferation (Oda et al., 2009). In addition, NR0B1 is overexpressed in cervical cancer and promotes cancer cell proliferation *via* the Wnt/β-catenin pathway (Liu et al., 2018). It is also overexpressed in pediatric adrenocortical tumors (de Sousa et al., 2015), ovarian cancer (Abd-Elaziz et al., 2003), breast cancer (Conde et al., 2004), endometrial cancer (Saito et al., 2005), and prostate cancer (Tong et al., 2014). Interestingly, NR0B1 was down-regulated in hepatocellular carcinoma tissues and cell lines and overexpression of NR0B1 could inhibit cell proliferation (Jiang et al., 2014), indicating that it may be a tumor suppressor in hepatocellular carcinoma. CREB3L3, also called CREB-H, was originally identified as a TF specifically expressed in the liver (Omori et al., 2001). The role of CREB3L3 in the liver is mainly related to triglyceride metabolism (Lee et al., 2011). A previous study has shown that loss of CREB3L3 function in hepatocellular carcinoma might contribute to the occurrence and/or progression of cancer (Chin et al., 2005). These results were consistent with our study that low expression of NR0B1 and CREB3L3 was associated with a worse prognosis of ACC patients. However, their potential roles in ACC have not been investigated.

CENPA is a histone-H3 variant that regulates cell division by establishing kinetochore assembly and ensuring proper centromere segregation and is associated with cancer progression (Athwal et al., 2015; Vardabasso et al., 2014). CENPA expression level is a potential biomarker of poor prognosis in cancer patients (Sun et al., 2016). FOXM1 and E2F2 are the upstream regulators of CENPA and play critical roles in cell cycle progression and tumorigenesis (Nakahata et al., 2014; Wang et al., 2011). Both TFs can potentially bind to the CENPA promoter sequence indicating that they regulate CENPA transcription (Grant et al., 2013). Previous studies have correlated CENPA with poor prognosis in ACC patients and identified E2F2 as an ACC-related TF (Xia et al., 2019; Zhang et al., 2013). Although few studies have associated CREB3L3, MYBL2, HOXC11, ZIC2, ZNF282, DNMT1, TCF2, FLK4, and KLF6 with ACC progression (DiFeo, Martignetti & Narla, 2009; Jain, Rechache & Kebebew, 2012), there is no evidence linking FOXM1 to ACC. We found that high levels of CREB3L3 and NR0B1 were correlated with good prognosis, while that of other TFs were correlated with poor prognosis in the ACC patients. The tumor stage was correlated with the OS of patients in the training set but not in the validation set. Furthermore, the 13-TF signature was an independent prognostic factor in both TCGA and GEO datasets.

The GSEA results showed that G2M-CHECKPOINT and E2F-TARGET were significantly enriched in the high-risk group. The G2/M checkpoint is frequently impaired in cancer cells, which promotes genomic instability and tumorigenesis (Lobrich & Jeggo, 2007). Since the E2F transcription factors regulate DNA replication and are aberrantly expressed in almost all human cancers (Johnson & Schneider-Broussard, 1998), targeting E2Fs could be a generic approach in anti-cancer treatment.

To the best of our knowledge, this report is the first to investigate the cancer-specific TFs and their association with clinical outcomes in ACC patients. The 13-TF signature showed accurate predictive ability and is a promising prognostic biomarker for clinical applications. However, the *in-silico* results were not validated by PCR or Western blotting. Future studies should focus on validating these survival-related TFs through molecular and functional assays, and determine the mechanistic basis.

## Abbreviations

ACC: adrenocortical carcinoma
TF: transcription factor
TCGA: The Cancer Genome Atlas
GEO: Gene Expression Omnibus
ROC: Receiver Operating Characteristic
OS: overall survival
CCLE: Cancer cell line encyclopedia
CREB3L3: cAMP-responsive element-binding protein 3-like 3
NR0B1: nuclear receptor subfamily 0, group B, member
CENPA: centromere protein-A
FOXM1: Forkhead Box M1
E2F2: E2F transcription factor 2
MYBL2: v-myb myeloblastosis viral oncogene homolog (avian)-like 2
HOXC11: homeobox C11
ZIC2: Zic family member 2
ZNF282: zinc finger protein 282
DNMT1: DNA methyltransferase
TCF3: transcription factor 3
ELK4: ETS transcription factor ELK4
KLF6: Krüppel-like factor 6
GSEA: Gene Set Enrichment Analysis

## Additional Information and Declarations

### Data Availability

The following information was supplied regarding data availability: All the raw data is available at TCGA (search term: ACC) and GEO: GSE19776.
